# Product distribution in the silicatein-catalysed synthesis of polydimethylsiloxane

**DOI:** 10.1039/d6cy00067c

**Published:** 2026-04-02

**Authors:** Yuqing Lu, Lu Shin Wong

**Affiliations:** a Manchester Institute of Biotechnology, University of Manchester 131 Princess Street Manchester M1 7DN UK l.s.wong@manchester.ac.uk; b Department of Chemistry, University of Manchester Oxford Road Manchester M13 9PL UK

## Abstract

Biocatalytic polymerisations of monomeric siloxane precursors to polysiloxane “silicone” polymers were recently reported. This study further investigates the polymerisation and depolymerisation of poly(dimethylsiloxane) from the monomeric precursor dimethyldimethoxysilane, catalysed by recombinant enzyme silicatein-α. Here, product distributions were systematically examined to assess the reaction outcome over time, and to deduce the pathways by which both linear polymers and small cyclic oligomers were produced. The results showed that short-chain oligomers were the predominant products at the early stage of the reaction, which subsequently polymerised into longer linear chains within 48 hours, reaching *Z*-averaged molecular weights of approximately 2000 Da. As the reaction progressed, the long-chain polymers underwent partial degradation into small amounts of cyclic oligomers under enzyme-catalysed conditions. However, the cyclic species did not undergo ring-opening polymerisation to linear chains under the same conditions. The system appeared to reach an equilibrium between polymer growth and degradation after 96 hours. These findings provide a foundation for further exploration of silicatein-catalysed polymerisation and highlight its potential for developing strategies for the sustainable synthetic manipulation of silicone polymers.

## Abstract 2

Silicatein-catalysed polymerisation pathways for poly(dimethyl)siloxane (PDMS) synthesis.

## Graphical abstract

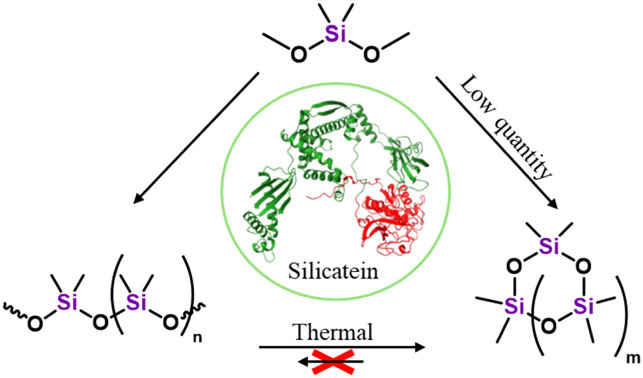

## Introduction

Polydimethylsiloxane (PDMS) is a highly versatile silicone polymer with broad applications in the electronics, construction and biomedical sectors, as well as in consumer products, due to its unusual combination of properties including thermal stability, biocompatibility and flexibility.^1–3^ Driven by the growing demand in these sectors, the global production of silicone-based products has been continuously increasing with the global silicone market size estimated be growing at 6% *per annum* to 2030.^4^ However, the conventional industrial production of PDMS through the hydrolysis and condensation of dimethyldichlorosilane presents several drawbacks. One issue is the generation of hydrochloric acid as a byproduct, which necessitates dedicated engineering measures for handling and disposal,^2^ leading to increased operational costs and environmental footprint.

Biocatalytic approaches have been explored in the synthetic manipulation of siloxanes. Here, enzyme-catalysed processes present a promising alternative by operating under milder conditions, which may lead to reduced energy consumption. The earliest examples of PDMS formation from monomeric dialkoxydimethylsilane precursors employed *Rhizopus delemar* lipase^5^ or *Suberites domuncula* silicatein^6^ as the biocatalyst under aqueous conditions, but only resulted in short PDMS chains (<10-mers). In parallel, the formation of small molecule disiloxanes by the condensation of their corresponding trialkylalkoxysilanes has also been demonstrated with hydrolytic enzymes such as trypsin, various lipases and phytases,^7–9^ though their conversions were modest; and in some cases later found to be non-specific (*i.e.* independent of the active site).^10^

More recent investigations of *Suberites domuncula* silicatein-α (Silα, the most abundant isoform of this enzyme) subsequently showed that Si–O bond condensations of small molecule silyl ethers could be achieved with high conversions in some cases by carrying out the reactions in non-aqueous media (*e.g.* octane, toluene) and elevated temperatures (≥75 °C).^11,12^ Work with this enzyme was subsequently extended to demonstrate the polymerisation of PDMS from dialkoxysilane precursors under similar conditions.^13^ Here, the formation of larger polymers (>20-mers) was achieved, alongside small quantities of cyclic oligomers (*e.g.* trimers, tetramers and pentamers; commonly referred to as “D3”, “D4”, and “D5”; respectively).

However, the reaction pathways by which the linear and cyclic polymers were produced from the monomeric precursors, and their potential interconversion, under these reaction conditions were unclear. Hence, this study aimed to investigate the relative contributions of the individual reaction steps in the silicatein-catalysed synthesis of PDMS.

## Results and discussion

### Product distribution during the polymerisation of dimethyldimethoxysilane

To investigate the evolution of product distributions, an extended time-course experiment was conducted over 120 hours under previously reported conditions (75 °C in toluene), using dimethoxydimethylsilane (DMDMS) as a readily available and non-hazardous precursor. As before, the catalyst employed was TF-Silα-Strep, a recombinant Silα consisting of an N-terminal trigger factor fusion and a C-terminal Strep-tag II.^14^ This enzyme was used in its lyophilised form, in a matrix of potassium phosphate and crown ether, which has previously been shown to facilitate siloxane condensation under the reaction conditions used in the present study.^12,13,15^ Reaction mixtures were sampled every 12 hours and analysed by GC and MALDI-MS to quantify substrate consumption, the formation of cyclic oligomers, and assess the molecular weight distribution of the linear polymers.

**Fig. 1:**
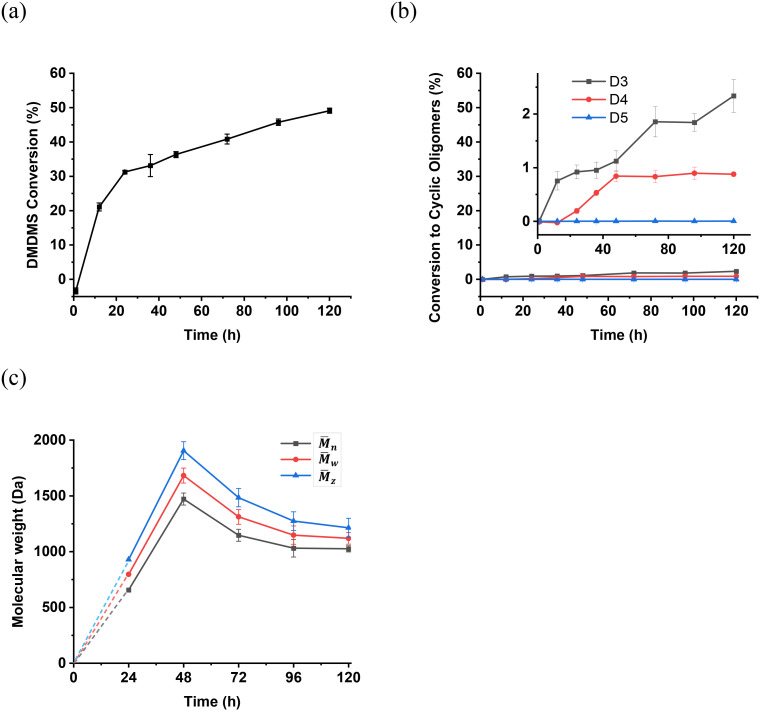
Plots of (a) percentage consumption of DMDMS in the enzymatic reaction, (b) percentage conversion to cyclic oligomers, and (c) molecular weights of enzymatically produced linear polymers over time. Numerical data use to produce these graphs are provided in Table S1, and were measured by GC-MS and MALDI-MS (see experimental section). Error bars represent the standard error of the mean from technical triplicates.

GC analysis revealed an initial rapid consumption of DMDMS, reaching 31% conversion within 24 hours in the presence of the enzyme (Fig. 1a). However, the rate of conversion slowed thereafter, approaching a final conversion of around 50% at 120 hours. In comparison, the purely thermal reaction where the enzyme was omitted resulted in a final conversion of 19% after this time (Table S1, see further discussion below). Less than 3% of the precursors were converted to cyclic oligomers in the enzymatic reaction (Fig. 1b), and only trace amounts were found when the enzyme was omitted (<1%, Table S1); implying that the majority of the substrate was converted into linear polymers.

**Fig. 2:**
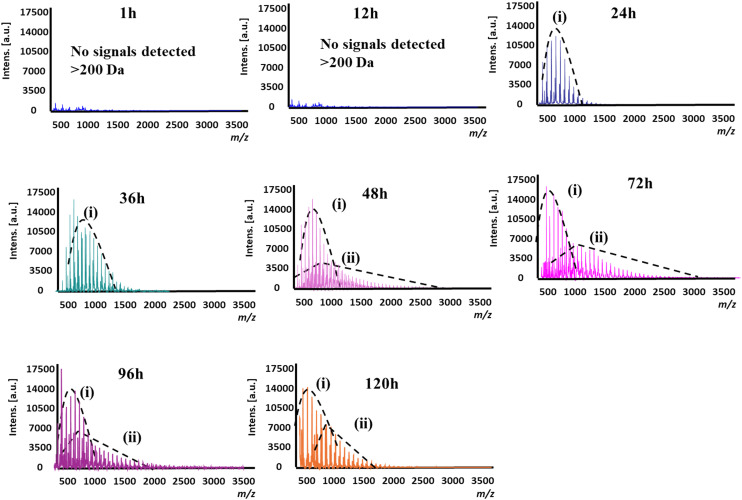
MALDI mass spectra of the products from the enzymatic polymerisation reactions of DMDMS over 1–120 hours, with population distributions indicated.

The MALDI-MS spectra showed no high molecular weight polymers in the first 12 hours of the reaction (Fig. 2). However, since approximately 24% of the substrate had already been consumed at this time (Fig. 1a), it appears likely that the predominant products in the early stage of the reaction are short-chain polymers, which are not detectable by MALDI-MS. Larger polymers with number-averaged molecular weight (*M̄*_n_) ∼550 Da were detected after 24 hours and continued to increase in size between 24 and 48 hours, suggesting that these short-chains gradually extended into larger polymers during the course of the reaction. After 48 hours, two distinct polymer distributions are observed (denoted as ‘i’ and ‘ii’ in Fig. 2, Table 1). This dual distribution is consistent with previous findings, where the polymers of lower molecular weight were the result of thermally-driven non-enzymatic reactions (*i.e.* they are present in the negative control where the enzyme is omitted, Table 1, Fig. S1 in SI), and the larger polymers were the result of enzyme-catalysed polymerisation.^13^

**Table 1:** Calculated average molecular weights of the polysiloxanes found in the polymerisation reactions. ‘i’ and ‘ii’ refer to the two population distributions of polymers observed in Fig. 2. n.d. = not determined, no polymer detected. *M̄*_n_, *M̄*_w_, and *M̄*_z_ represent the number-average, weight-average, and *Z*-average molecular weights, respectively

Time (hours)	*M̄*_n_		*M̄*_w_		*M̄*_z_		Polydispersity	
	Non-enzymatic	Enzymatic	Non-enzymatic	Enzymatic	Non-enzymatic	Enzymatic	Non-enzymatic	Enzymatic
1	n.d.	n.d.	n.d.	n.d.	n.d.	n.d.	n.d.	n.d.
12	n.d.	n.d.	n.d.	n.d.	n.d.	n.d.	n.d.	n.d.
24	482	(i) 657	571	(i) 797	657	(i) 929	1.18	(i) 1.21
36	545	(i) 960	645	(i) 1114	746	(i) 1271	1.18	(i) 1.16
48	703	(i) 782	721	(i) 814	740	(i) 846	1.02	(i) 1.04
		(ii) 1471		(ii) 1682		(ii) 1906		(ii) 1.14
72	715	(i) 837	743	(i) 857	774	(i) 876	1.04	(i) 1.03
		(ii) 1147		(ii) 1312		(ii) 1484		(ii) 1.14
96	782	(i) 713	803	(i) 740	825	(i) 768	1.03	(i) 1.04
		(ii) 1031		(ii) 1148		(ii) 1274		(ii) 1.11
120	688	(i) 677	719	(i) 699	754	(i) 720	1.05	(i) 1.04
		(ii) 1025		(ii) 1119		(ii) 1213		(ii) 1.09

However, beyond 48 hours, a decrease in the molecular weight of the heavier polymer fraction (ii in Table 1 and Fig. 2) was observed, with the molecular weight values apparently stabilizing after 96 hours (Table 1, Fig. 1c). These results show that these larger linear polymers also undergo depolymerisation, potentially through enzyme-mediated processes (see below), eventually approaching an equilibrium between polymer growth and degradation after this time.

**Fig. 3:**
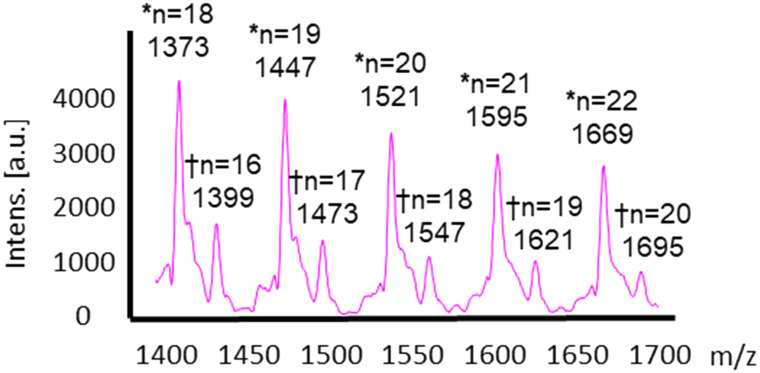
Magnification of the MALDI mass spectrum from the enzymatic polymerisation reactions of DMDMS after 48 hours. The ion series marked * and † correspond to [HO(SiMe_2_O)_*n*_H + Na]^+^ and [HO(SiMe_2_O)_*n*_H + K_2_HPO_4_ + Na]^+^, respectively.

For the larger polymers formed by enzyme catalysis, an analysis of the individual peak *m*/*z* values in the mass spectrum showed that these polymers were hydroxy terminated, whereby ions corresponding to the series [HO(SiMe_2_O)_*n*_H + Na]^+^ and [HO(SiMe_2_O)_*n*_H + K_2_HPO_4_ + Na]^+^ were observed (Fig. 3). Here, the latter ion series was observed due to the potassium phosphate in the lyophilisation matrix. No ions corresponding to the mono- or dimethoxy end-capped polymers were identified. This result suggests that enzymatic hydrolysis of the methoxy groups may be the main driver for polymerisation, which is consistent with the fact that initial hydrolysis to form a silanol is the rate-limiting step in polysiloxane polymerisation.^16^

**Scheme 1:**
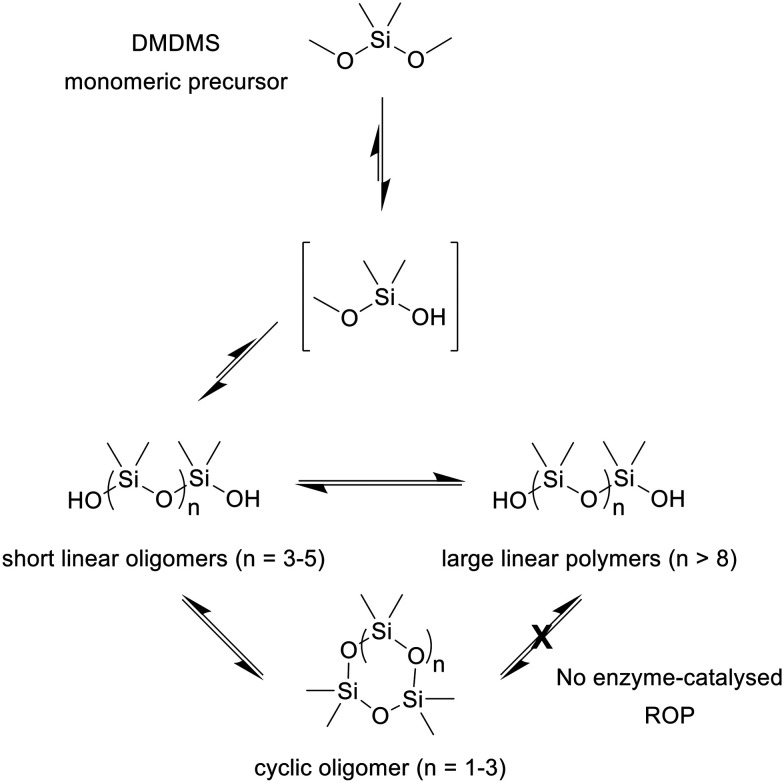
Proposed pathways to the silicatein-catalysed formation of polysiloxanes. Reaction conditions: TF-Silα-Strep (cat.), toluene, 75 °C.

In principle, the formation of these linear and cyclic oligomers could proceed through several pathways (Scheme 1). The monomers may initially hydrolyse and form short oligomers that subsequently either cyclise, or further elongate to larger linear polymers. These longer polymers could also arise from the ring-opening polymerisation (ROP) of the cyclic oligomers. Furthermore, any linear polymers may also depolymerize into smaller linear oligomers or monomers, or directly into the cyclic oligomers by backbiting (*i.e.* the reverse of ROP). Thus, a series of further experiments were conducted to elucidate the contributions and feasibility of each pathway.

### Silicatein-catalysed ring-opening polymerisation

First, the ability of silicatein to catalyse the ROP of cyclic oligomers to linear PDMS was examined. To favour ROP, D3 was employed as a substrate due to its higher reactivity compared to larger cyclic oligomers.^17^ Here, D3 was subjected to the same reaction conditions (75 °C in toluene) for 48 hours and the reaction products were analysed by MALDI-MS and GC-MS. Experiments were also carried out where the enzyme was omitted, as the negative controls.

In all cases, no polymers were found in the MALDI-MS analysis under all conditions (SI Fig. S2). Furthermore, GC analysis demonstrated that the D3 remained unconsumed in both the control and biocatalytic reactions (SI Fig. S3). These results therefore show ROP of D3 does not occur under these reaction conditions, and that D3 is not accepted by the enzyme as a substrate. Since D3 contains strained Si–O bonds that would have an intrinsically higher reactivity than the analogous bonds in unconstrained DMDMS, it suggests that the lack of enzyme-catalysed reactivity is due to inability of the substrate to access the active site of the enzyme. This is unsurprising since computational calculations show that D3 has twice the molecular size of DMDMS (Connolly solvent excluded volume of 213 *vs.* 119 Å^3^). These results also imply that any of the oligomers present in these reactions were not produced by enzyme-catalysed backbiting.

### Depolymerisation of polysiloxane

**Fig. 4:**
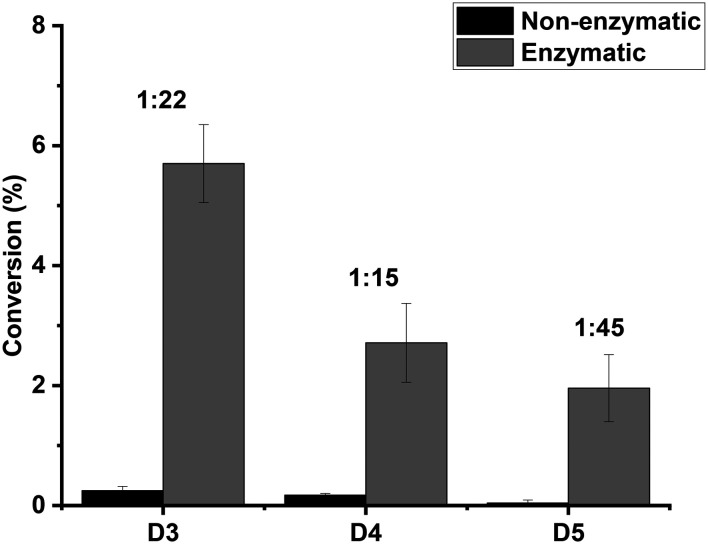
Graph of percentage conversion to cyclic oligomers in the enzymatic and non-enzymatic depolymerisation of HO-PDMS after 48 hours. The ratios of the enzymatic to non-enzymatic reaction are shown above each column pair. The error bars represent the standard error of mean from triplicate experiments.

To elucidate whether the enzyme can catalyse the depolymerisation of the linear polymer, hydroxy-terminated poly(dimethylsiloxane) (HO-PDMS, *M̄*_n_ ∼550) was treated under the same reaction conditions (75 °C in toluene for 48 hours). The reaction mixture analysed by GC-MS and any newly observed chromatographic peaks and their corresponding mass spectra were matched against authentic standards of D3, D4 and D5. Here, it was found that TF-Silα-Strep did indeed catalyse the formation of cyclic oligomers from HO-PDMS, with D3 being formed in the largest relative proportion, followed by D4 and D5 (Fig. 4). In comparison, the negative control reaction gave only trace amounts of cyclic oligomers.

If enzyme-catalysed backbiting is excluded, this result suggests that TF-Silα-Strep catalyses the cleavage of linear PDMS into short oligomers or monomers, from which the cyclic species are subsequently formed. The small amounts of cyclosiloxanes detected in the negative control indicate that the linear polymer can nevertheless undergo gradual degradation *via* thermally activated backbiting^18,19^ in the absence of the enzyme, albeit only at trace levels under the relatively low temperatures utilised here. Regardless of the pathway to their formation, the preferential formation of D3 despite its ring strain was unexpected. This result may reflect kinetic control over the cyclisation process, as previously reported under non-aqueous conditions where smaller rings form more rapidly than their thermodynamically favoured counterparts.^20^

Taken together, these results show that under the reaction conditions investigated here, linear polymers represent the dominant product, while cyclic species are formed as minor side products. The enzyme appears to primarily promote the linear extension of chains from monomeric precursors, since it does not catalyse ROP and thus presumably also not backbiting (Scheme 1). The formation of longer polymer chains in the presence of TF-Silα-Strep is likely due to its acceleration of condensation reactions (*i.e.* the largest linear polymers are a kinetic product). In contrast, non-enzymatic background reactions (thermally-driven backbiting and cyclisation of the small oligomers), and the enzyme-catalysed depolymerisation, proceed more slowly. This interpretation is consistent with the observed increase in weight-average and *Z*-average molecular weights, along with a broader polydispersity, in enzymatic reactions. Once the growing polymer chains reach a defined size, further elongation is limited due the depolymerisation reactions that are occurring in parallel, eventually resulting in the equilibrium mixture found at the end of the reaction (beyond ∼96 hours).

These results share similarities with previous work on esterase-catalysed polyester condensations where the primary products are linear polyesters, while cyclic by-products occasionally form depending on reaction conditions.^21–24^ In esterase-catalysed polymerisation, high molecular weight polyesters also typically reach an equilibrium state, beyond which further chain elongation is restricted. This equilibrium is influenced by reaction conditions, enzyme specificity, and polymer chain stability.

## Conclusion

In summary, this study employed TF-Silα-Strep and DMDMS as the model biocatalyst and substrate, respectively, to investigate the evolution of biocatalytic polysiloxane formation. These results confirm earlier preliminary studies^13^ showing that enzyme can catalyse the formation of long-chain linear polysiloxanes from DMDMS in anhydrous solvent at elevated temperatures.

Further analyses revealed a rapid consumption of the substrate over the first 24 hours, leading to the formation of short- to medium-length linear polymers. Enzymatic catalysis then promoted further polymer growth, yielding higher molecular weight linear polysiloxanes, whereas non-enzymatic reactions only resulted in low molecular weight oligomers. Although polymer formation can occur purely thermally (without enzyme), the polymers that are formed are short and conversions are low. Thus, the results here show that enzyme-catalysed polymerisation is the dominant pathway (majority of product) under the conditions investigated here.

These linear polymers undergo some enzymatic degradation upon prolonged reaction (>48 h) but the system eventually reaches a steady state, with the polymer size stabilising at the presumed thermodynamic equilibrium (*i.e.* rate of polymerisation approximates rate of depolymerisation from this time point onwards).

In the course of this reaction small amounts of cyclic oligomers are produced in a process that is facilitated by the enzyme. However, their formation is probably not due to enzyme-catalysed backbiting, since the reverse transformation (*i.e.* ROP of cyclic oligomers into linear polymers) is not catalysed by the enzyme, as evidenced by the fact that TF-Silα-Strep rejects D3 as a substrate.

The similarities between silicatein-catalysed DMDMS polymerisation and esterase-catalysed polyester formation suggest that approaches commonly used to regulate polymer size and reduce cyclic by-products in polyester synthesis, such as modifying reaction conditions, enzyme concentration, and substrate availability, could also be effective in optimising silicatein-mediated polymerisation. Since methanol is a byproduct of these polymerisations, incorporation of methods for its continuous removal may also be beneficial.

Overall, this study provides further insights into the biocatalytic conversion of dimethyldimethoxysilane to PDMS, and may pave the way for future efforts to develop sustainable routes to PMDS production. Some results here also suggest the intriguing possibility harnessing biocatalysis for the degradation and recycling of long-chain polymers.

## Experimental

### Materials and methods

All reagents, authentic product samples and solvents were purchased from either Sigma Aldrich or Fisher Scientific in their highest available purity. The commercially sourced DMDMS and D3 were found to be homogenous by MALDI-MS and GC-MS and were used without further purification. HO-PDMS was analysed prior to use and any cyclic oligomers that were found were deducted from the conversion calculations in subsequent reactions. The TF-Silα-Strep enzyme was heterologously produced in *E. coli* and prepared in a lyophilised form as previously described.^12^ The condensation reactions were performed in crimp-sealable 8 mm vials (Chromacol C4008-741) using an Eppendorf Thermomixer 5350 for heating and shaking.

### Enzymatic polymerisation reactions

For each experiment, a 100 μL solution of the desired substrate (0.7 M in toluene) was added to a vial containing 0.5 mg of lyophilised enzyme preparation. The vials were crimp-sealed and heated at 75 °C while shaking at 650 rpm for the desired reaction time. For the control reaction, the lyophilised enzyme was omitted and replaced with only the additives (potassium salts and 18-crown-6) in lyophilised form. All reactions were performed in triplicate.

### MALDI-MS analysis

After the designated reaction time, the vials were removed from heating and allowed to cool to room temperature. The reaction mixtures were then withdrawn using a 1 mL syringe and transferred to 1.5 mL centrifuge tubes. Each sample was diluted with 100 μL of pentane and centrifuged at 17 000*g* for 10 minutes to remove any insoluble material. A 10 μL aliquot of the supernatant was mixed with 10 μL of matrix solution containing 10 mg mL^−1^ of 2,5-dihydroxybenzoic acid in 50% acetonitrile, 50% H_2_O, 0.1% TFA. Then, 2 μL of this mixture was applied on to a MALDI-MS target plate and allowed to dry under ambient conditions. MALDI-MS analyses were conducted with a Bruker Biotyper Sirius and the mass spectra were calibrated using the ACTH peptide fragment 18-39 and oxidised insulin B chain (both sourced from Sigma-Aldrich).

### GC-MS analysis

Following MALDI-MS sample preparation, the remaining supernatant was further diluted with pentane to a final volume of 7.2 mL. These solutions were transferred to 15 mL centrifuge tubes and centrifuged at 17000 g for 10 minutes to remove any residual insoluble material. A 1 mL aliquot of each supernatant was then transferred into a clean vial for GC-MS analysis.

For quantification of the substrate and cyclic oligomer conversion, standard calibration curves were prepared using authentic standards of DMDMS, D3, D4 and D5 in the same toluene/pentane solvent mixture (SI Fig. S4). The prepared standard solutions and reaction samples were analysed under identical conditions using the previously reported GC-MS method.^12,13^ Percent conversions were calculated by comparing the GC-MS peak areas of the substrate or cyclic oligomers in the reaction samples against the respective calibration curves.

### Enzymatic depolymerisation reactions

A 100 μL solution of the polymer solution (10 μL HO-PDMS, *M̄*_n_ = ∼550 dissolved in 90 μL toluene) was added to a vial containing 0.5 mg of lyophilised enzyme. The vials were crimp-sealed and heated at 75 °C while shaking at 650 rpm for 48 hours. The vials were then removed from heating and allowed to cool to room temperature. The reaction mixtures were then withdrawn using a 1 mL syringe and transferred to 1.5 mL centrifuge tubes. Each sample was diluted with 1 mL of acetonitrile and centrifuged at 17 000*g* for 10 minutes to remove any insoluble material. A 1 mL aliquot of each supernatant was then transferred into a clean vial for GC-MS analysis. For the control reaction, the lyophilised enzyme was omitted and replaced with only the additives (potassium salts and 18-crown-6) in lyophilised form. All reactions were performed in triplicate.

### Calculations of molecular volume

Calculations were carried out on Chem3D version 18.0.0.231 (PerkinElmer Informatics). The chemical structure drawn in ChemDraw *.cdxml format were imported into the Chem3D application. Molecular dynamics (MM2) calculations are then carried out to iteratively minimise energy to a minimum RMS gradient of 0.01. The Connolly solvent excluded volume was subsequently calculated using a probe radius of 1.4 Å (nominally the size of water molecules).

## Author contributions

Conceptualization, L. S. W.; methodology, Y. L.; formal analysis, Y. L. and L. S. W.; investigation, Y. L.; resources and data curation, Y. L.; writing – original draft preparation, Y. L.; writing – review & editing, Y. L. and L. S. W.; supervision, L. S. W.; project administration, L. S. W.

## Conflicts of interest

The authors declare no conflict of interest. The funders had no role in the design of the study, in the collection, analyses, or interpretation of data, in the writing of the manuscript, or in the decision to publish the results.

## Supplementary Material

CY-016-D6CY00067C-s001

## Data Availability

The numerical data used to produce the results herein are available on Figshare at https://doi.org/10.6084/m9.figshare.30746669. Supplementary information (SI) is available. See DOI: https://doi.org/10.1039/d6cy00067c.
