# Supplementary material for: Product distribution in the silicatein-catalysed synthesis of polydimethylsiloxane
Source: Catal Sci Technol. 2026 Apr 2;16(9):3183–9. doi: 10.1039/d6cy00067c (PMC13070288; doi:10.1039/d6cy00067c)
Supplement: CY-016-D6CY00067C-s001 [file CY-016-D6CY00067C-s001.pdf]

Supplementary Information (SI) for Catalysis Science & Technology.
This journal is © The Royal Society of Chemistry 2026

Supplementary Information

Product Distribution in the Silicatein-Catalysed Synthesis of Polydimethylsiloxane

Yuqing Lu,[a,b] Lu Shin Wong[a,b]

[a] Manchester Institute of Biotechnology, University of Manchester, Manchester M1 7DN, United Kingdom;

[b] Department of Chemistry, University of Manchester, Manchester M13 9PL, United Kingdom

* correspondence: l.s.wong@manchester.ac.uk

Supplementary Results

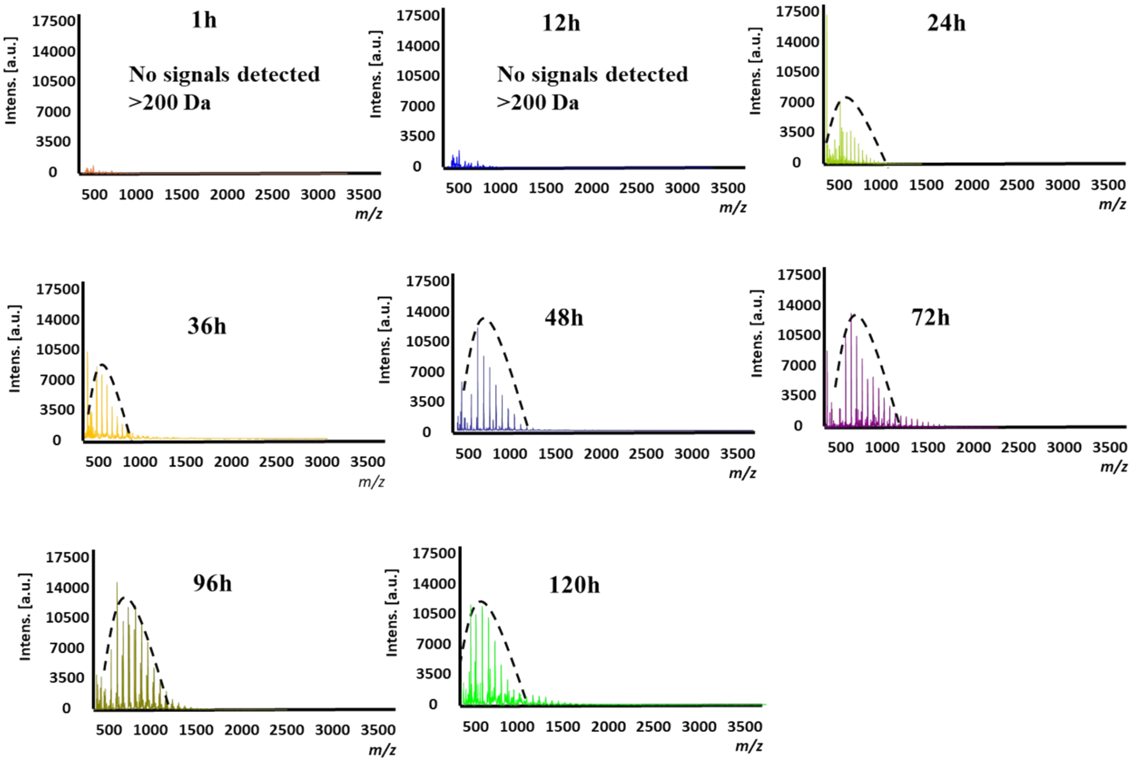

Figure S1. MALDI mass spectra of the products from the polymerisation reactions of DMDMS over 1–120 hours in non-enzymatic reactions.

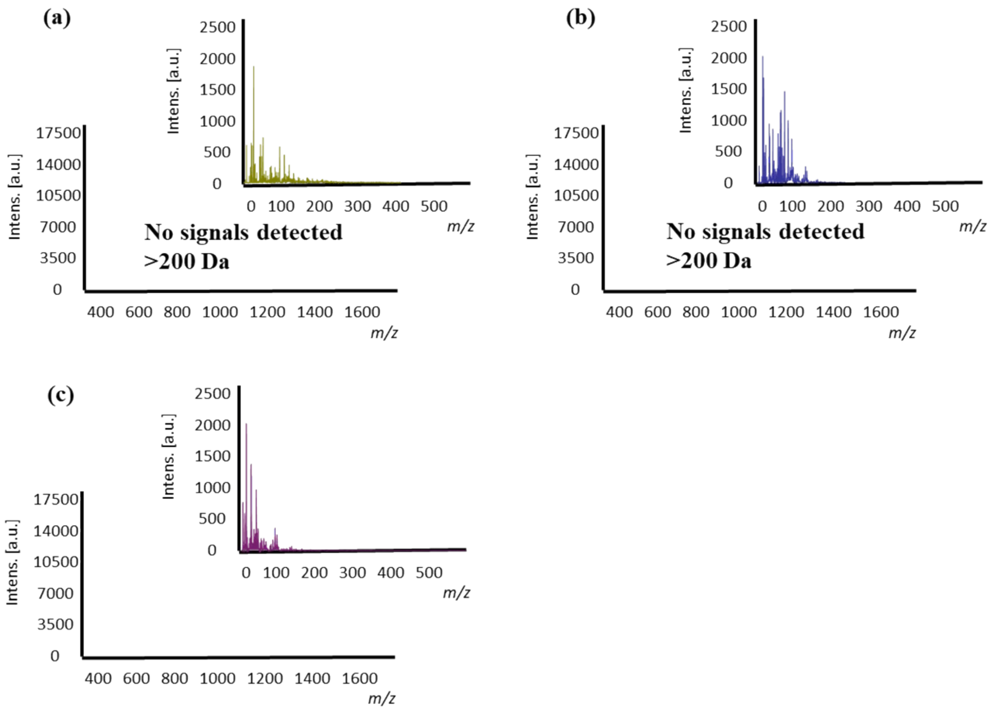

Figure S2. MALDI mass spectra for D3 ring-opening polymerisation after 48 hours. The inset shows the mass spectra in the low *m/z* range (0–500) for: (a) the substrate prior to reaction, (b) the control reaction (enzyme omitted), and (c) the fully constituted reaction.

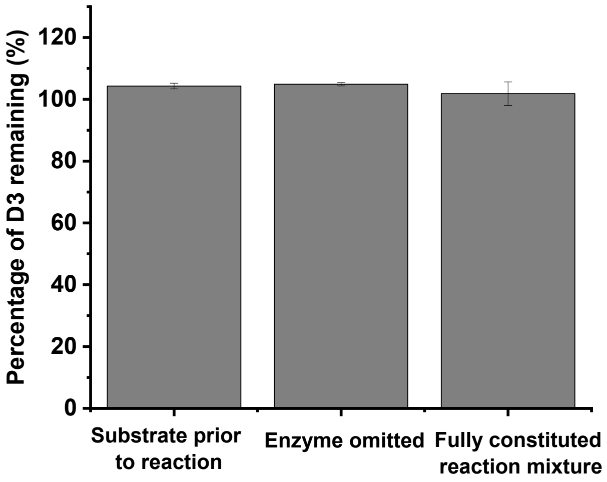

Figure S3. The percentage of D3 remaining after ring-opening polymerisation after 48 hours. The error bars indicate standard error of the mean from technical triplicates.

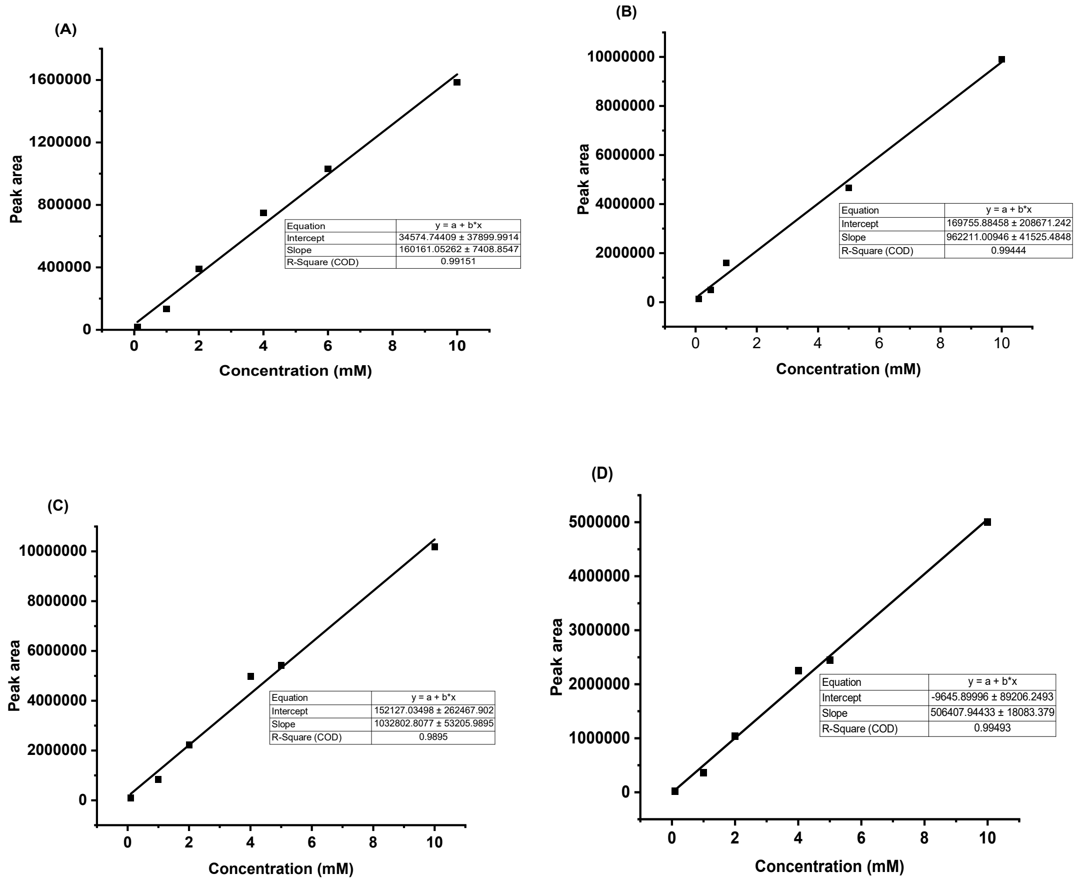

Figure S4. GC Calibration plots for product quantification. Calibration graph of peak area against concentration of dimethyldimethoxysilane (A), D3 (B), D4 (C), D5 (D).

Table S1. Percentage of consumption of DMDMS and conversion to cyclic siloxanes over time. The net conversion was calculated by the difference between the enzymatic reaction conversion and the non-enzymatic reaction conversion.

Compound	Time (h)	Consumption (%)		
		Enzymatic	Non-enzymatic	Net
DMDMS	1	~0	~0	~0
	12	21.0±1.1	8.6±1.4	12.4±1.8
	24	31.2±0.4	9.3±1.0	21.9±1.0
	36	33.1±3.2	11.1±1.4	22.0±3.5
	48	36.3±0.8	13.1±0.8	23.2±1.1
	72	40.8±1.4	14.7±1.0	26.1±1.7
	96	45.7±0.9	17.1±0.9	28.6±1.3
	120	49.0±0.7	19.0±0.3	30.0±0.8
		Conversion (%)		
D3	1	~0	~0	~0
	12	0.2±0.2	~0	0.20±0.2
	24	0.6±0.1	~0	0.6±0.1
	36	0.9±0.2	0.03±0.05	0.9±0.2
	48	1.3±0.2	0.2±0.07	1.1±0.2
	72	2.0±0.3	0.1±0.06	1.9±0.3
	96	2.2±0.2	0.3±0.04	1.9±0.2
	120	2.8±0.4	0.5±0.1	2.3±0.4
D4	1	~0	~0	~0
	12	~0	~0	~0
	24	0.22±0.07	0.021±0.02	0.19±0.05
	36	0.68±0.02	0.15±0.03	0.53±0.05
	48	1.07±0.06	0.23±0.05	0.84±0.1
	72	1.04±0.1	0.20±0.007	0.83±0.1
	96	1.11±0.07	0.21±0.04	0.89±0.1
	120	1.12±0.02	0.25±0.05	0.88±0.04
D5	1	<0.1%	<0.1%	<0.1%
	12	<0.1%	<0.1%	<0.1%
	24	<0.1%	<0.1%	<0.1%
	36	<0.1%	<0.1%	<0.1%
	48	<0.1%	<0.1%	<0.1%
	72	<0.1%	<0.1%	<0.1%
	96	<0.1%	<0.1%	<0.1%
	120	<0.1%	<0.1%	<0.1%

Supplementary Experimental Methods

Calculation methods:

Consumption of DMDMS $(\%) = \frac{C_{initial} - C_{final}}{C_{initial}} \times 100\%$

where:

- $C_{initial}$: The concentration of DMDMS added at the start of the reaction
- C_{final} : The concentration of DMDMS after reaction

Cyclic product conversion $(\%) = \frac{X_i \times n_{Di}}{n_{DMDMS,initial}} \times 100\%$

where:

- X_i : The number of $SiMe_2O$ units per cyclic species (e.g., 3 for D3 4 for D4, 5 for D5)
- n_{Di}: mol of each cyclic oligomer
- $n_{DMDMS,initial}$: The total number of moles of DMDMS added to the reaction.

The conversion of hydroxy-terminated PDMS to cyclic oligomers (e.g., D3, D4, D5):

Cyclic product conversion $(\%) = \frac{X_i \times n_{Di}}{n_{SiMe_2O,initial}} \times 100\%$

Where:

- X_i : The number of $SiMe_2O$ units in that species (3 for D3, 4 for D4, 5 for D5)
- n_{Di}: The number of moles of each cyclic species (e.g., D3, D4, D5)
- $n_{SiMe_2O,initial}$: The total initial number of moles of $SiMe_2O$ units in the HO-PDMS substrate
